# Brain Modulation by Electric Currents in Fibromyalgia: A Structured Review on Non-invasive Approach With Transcranial Electrical Stimulation

**DOI:** 10.3389/fnhum.2019.00040

**Published:** 2019-02-11

**Authors:** Filippo Brighina, Massimiliano Curatolo, Giuseppe Cosentino, Marina De Tommaso, Giuseppe Battaglia, Pier Carlo Sarzi-Puttini, Giuliana Guggino, Brigida Fierro

**Affiliations:** ^1^Dipartimento di Biomedicina, Neuroscienze e Diagnostica Avanzata (BIND), Università degli Studi di Palermo, Palermo, Italy; ^2^Department of Brain and Behavioral Sciences, University of Pavia, Pavia, Italy; ^3^IRCCS Mondino Foundation, Pavia, Italy; ^4^Unità di Neurofisiopatologia del Dolore, Dipartimento di Scienze Mediche di Base, Neuroscienze e Organi di Senso (SMBNOS), Università degli Studi di Bari Aldo Moro, Bari, Italy; ^5^Dipartimento di Scienze Psicologiche, Pedagogiche, dell’Esercizio Fisico e della Formazione, Università degli Studi di Palermo, Palermo, Italy; ^6^Department of Rheumatology, Luigi Sacco University Hospital, Milan, Italy; ^7^Dipartimento Biomedico di Medicina Interna e Specialistica (DIBIMIS), Università degli Studi di Palermo, Palermo, Italy

**Keywords:** non-invasive brain stimulation (NIBS), transcranial electrical stimulation (tES), fibromyalgia (FM), tDCS — transcranial direct current stimulation, tRNS (transcranial random noise stimulation)

## Abstract

Fibromyalgia syndrome (FMS) is a complex disorder where widespread musculoskeletal pain is associated with many heterogenous symptoms ranging from affective disturbances to cognitive dysfunction and central fatigue. FMS is currently underdiagnosed and often very poorly responsive to pharmacological treatment. Pathophysiology of the disease remains still obscure even if in the last years fine structural and functional cerebral abnormalities have been identified, principally by neurophysiological and imaging studies delineating disfunctions in pain perception, processing and control systems. On such basis, recently, neurostimulation of brain areas involved in mechanism of pain processing and control (primary motor cortex: M1 and dorsolateral prefrontal cortex: DLPFC) has been explored by means of different approaches and particularly through non-invasive brain stimulation techniques (transcranial magnetic and electric stimulation: TMS and tES). Here we summarize studies on tES application in FMS. The great majority of reports, based on direct currents (transcranial direct currents stimulation: tDCS) and targeting M1, showed efficacy on pain measures and less on cognitive and affective symptoms, even if several aspects as maintenance of therapeutical effects and optimal stimulation parameters remain to be established. Differently, stimulation of DLPFC, explored in a few studies, was ineffective on pain and showed limited effects on cognitive and affective symptoms. Very recently new tES techniques as high-density tDCS (HD-tDCS), transcranial random noise stimulation (tRNS) and tDCS devices for home-based treatment have been explored in FMS with interesting even if very preliminary results opening interesting perspectives for more effective, well tolerated, cheap and easy therapeutic approaches.

## Introduction

Fibromyalgia syndrome (FMS) is a complex disorder where a widespread musculoskeletal pain (without a clear lesion basis) is associated with a great variety of symptoms including affective disturbances, central fatigue, cognitive dysfunction and even a particular skin reactivity to several chemical substances (Cassisi et al., 2008; Bazzichi et al., 2016). The manifestations of the disease are responsible for a consistent clinical burden that severely affects life quality of FMS patients. Indeed, aside pain, patients are disabled by sleep disturbances, anxiety and depression and by a complex cognitive dysfunctioning picture characterized by disturbance in attention, working memory and executive functions globally known as “fibrofog” often referred by the patients as a sense of confusion, slowing down and clumsiness that can severely impact the ability to effectively plan and perform daily activities (Tesio et al., 2015; Gelonch et al., 2016).

Despite intense research effort, especially in the last years, the pathophysiology of the disease remains to be clarified. However, principally thanks to electrophysiological and brain imaging techniques, some important advance has been obtained concerning the putative brain structural and functional abnormalities underlying the clinical manifestation of FMS. On such basis, great attention was pointed toward the role of central dysfunctional mechanisms in pathophysiology of FMS and the targets for research and potential new treatments moved to brain areas and networks involved in pain processing and control (prefrontal, insular and posterior cingulate regions) that can be accessed directly or through connected areas like motor cortex or dorsolateral prefrontal cortex. In this regard, particular interest has been raised by techniques able to perform effective modulation of brain areas through magnetic or electric currents applied to the scalp like transcranial magnetic and electrical stimulation (TMS and tES) (Hou et al., 2016; Zhu et al., 2017). TMS and tES are safe, non-invasive brain stimulation (NIBS) approaches that are able to modulate the activity of cortical areas inducing lasting effects that have been employed to investigate and treat neuropsychiatric diseases and pain disorders. Among NIBS techniques, tES seems particularly attractive as it is based on easy to use, quite cheap and small devices that appear suitable for patient’s self-use and home-based treatments (Lefaucheur et al., 2017). The potential therapeutic role of tES appears also relevant considering that most pharmacological and non-pharmacological treatments available are generally poorly effective or show only transitory efficacy.

Principal aim of the present paper was to perform a structured review concerning evidence on tES treatment for fibromyalgia with particular regard to transcranial directs stimulation (tDCS) that represents the most studied approach. We present also an overview on new tES techniques drawing perspective for future development. We discuss the results obtained in terms of effectiveness and safety, mentioning also the controversial aspect and raising suggestions for evaluating the real efficacy of the technique and for further therapeutic developments. The analysis of tES studies will be preceded by introductory notes on tES techniques and on pathophysiology of FMS with particular reference to area or network dysfunctions that could represent useful targets for neurostimulation.

## tES: Principles and Techniques

**FIGURE 1:**
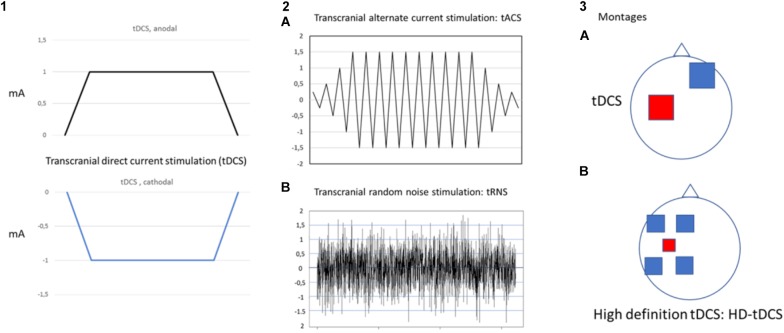
Different forms of transcranial electrical stimulation. mA (milliampere). Stimulating current can be: direct, continuous (transcranial direct current stimulation: tDCS) **(1)** that can be anodal or cathodal; alternate **(2)** with polarity changing a different frequency between anode and cathode at fixed (**2A** transcranial alternate current stimulation: tACS) or randomly changing frequency (**2B** transcranial random noise stimulation: tRNS). Different montages **(3)** can also be applied (anodal stimulation of M1 is exemplified in the picture). In classical tDCS **(3A)** anode is on the target and cathode in the reference area (contralateral supraorbital region); in the case of High definition-tDCS (HD-tDCS) **(3B)** anode (more smaller in size to increase focality) is positioned over the target area and is surrounded by four equally-spaced cathodes.

Transcranial electrical stimulation is a neurophysiological technique able to perform effective, safe, not invasive and painless brain stimulation in humans (Paulus, 2011). tES works through low amperage electric fields delivered through surface electrodes applied on the scalp. The first and the most common tES approach used works through direct currents: transcranial direct currents stimulation (tDCS) (Figure 1). Differently from TMS, tDCS is not able to trigger direct neuronal activation but rather exerts its effect through the polarization of the underlying neural cell membranes. Anodal currents induce neuronal depolarization increasing excitability and spontaneous neural firing while the reverse occurs with cathodal stimulation. tDCS can induce plastic effects that last after stimulation. In the seminal study by Nitsche and Paulus (2000), tDCS of motor cortex induced effects on motor evoked potential (facilitation by anodal and inhibition by cathodal currents) that remained for 5–10 min after stimulation. On such a basis, tDCS has been then applied over repeated stimulation sessions, in a manner similar to rTMS treatment protocols, with the aim to induce more lasting and effective modulation and showed promising results, in terms of efficacy and safety, for treatment of different chronic pain states and of many other neurological and psychiatric disorders (Nitsche et al., 2008; Lefaucheur et al., 2017). Recently, new tES approaches have been developed, potentially more effective and alternative to tDCS, to investigate and treat brain diseases. Among these, mention deserves high-definition tDCS (HD-tDCS), that increases the focality of stimulating currents, and transcranial alternating current stimulation (tACS), a technique using alternate currents delivered at different frequencies, with the aim to interact with the ongoing cortical neurons oscillations. HD-tDCS uses a particular electrodes’ arrangement with one target electrode (anode or cathode) much smaller than those used for tDCS, surrounded by a group of four equidistant electrodes with opposite polarity. This to get a more focal current flow of the required polarity over the target area, to have more focused, less diffuse and hypothetically more effective facilitatory or inhibitory effects. Therapeutic evidence of HD tDCS is till now poor and limited, in pain states, to fibromyalgia. Other even more preliminary applications concern tinnitus, aphasia, (Richardson et al., 2015; Lefaucheur et al., 2017) memory loss in MCI syndrome (Hampstead et al., 2017), epilepsy (Karvigh et al., 2017) and auditory hallucination in schizophrenic patients (Sreeraj et al., 2018).

**FIGURE 2:**
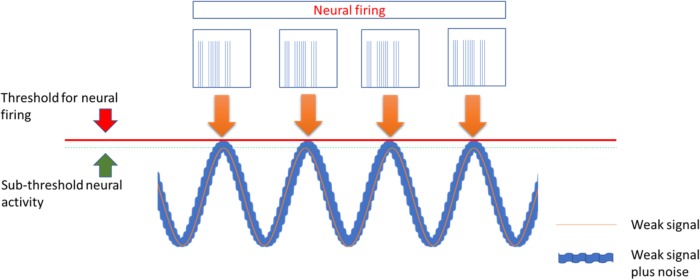
The stochastic Resonance, a phenomenon invoked to explain the effects of tACS and more in particular of tRNS. According to this principle a signal containing a high level of noise has more chance to increase excitability of neurons that are near to activation threshold making them to discharge.

Transcranial alternating current stimulation work through alternate, sinusoid currents that change polarity between electrodes at different stimulation frequencies. Currents can also be delivered at not fixed oscillating rate but with randomly changing frequencies across stimulation: transcranial random noise stimulation (tRNS) (Paulus, 2011; Fertonani and Miniussi, 2017) (Figure 1). Differently from tDCS, tACS induce none polarization effect and it should exert a modulatory interaction with the ongoing brain activity at a specific stimulation frequency. Such interaction, defined as entrainment, has been demonstrated in experimental animals (Reato et al., 2013) but also studies in humans showed effects on sensory and motor cortex suggesting entrainment (Hermann et al., 2013; Guerra et al., 2016). However, no study has so far explored potential effects of tACS for treatment of pain and other neuropsychiatric diseases. Differently from tACS that oscillate at fixed frequency, tRNS can induce a more generalized activation, thanks to the wide range of stimulation frequencies employed, based on the principle of stochastic resonance. According to this, a signal that is too weak to reach a certain threshold can be increased by adding noise (Fertonani and Miniussi, 2017) (Figure 2). In this way tRNS can favor the synchronization of nervous stimuli, through the amplification of neural sub-threshold activity. Motor cortex tRNS induced an effect stronger than anodal tDCS on cortical excitability in healthy subjects (Moliadze et al., 2014; Inukai et al., 2016), improved neuropathic pain in some case series (Alm and Dreimanis, 2013) and ameliorated pain and cognitive dysfunction in patients with multiple sclerosis (Palm et al. (2016).

## Pathophysiology of Fibromyalgia and Potential Targets for Neurostimulation

Pathophysiological mechanisms underlying the multifaceted clinical picture of fibromyalgia remain largely obscure. Recently, however, the application of new investigational approach able to better explore structural and functional brain changes has been successfully applied to the study of FMS revealing abnormalities that could at least in part account for the complex pain perception and processing dysfunction underlying the manifestations of the disease.

Fibromyalgia is considered a chronic pain syndrome characterized by an increased responsivity to painful stimuli. FMS patient have normal ability to perceive sensory stimuli but show reduced thresholds to pain (Lautenbacher and Rollman, 1997; Dadabhoy and Clauw, 2006). Such dysfunctions have been suggested to depend upon both central sensitization mechanisms and defective activity of the Diffuse Noxious Inhibitory Controls (DNIC) pathways that are involved in the inhibitory modulation of nociceptive input (Lee et al., 2011; Ceko et al., 2012).

According to this, electrophysiology and functional magnetic resonance imaging, revealed an abnormally increased reactivity of cortical regions of the pain network involved, at different extent, in pain perception and processing like medial prefrontal and insular areas, sensory and motor cortex and cerebellum, while a decreased activity and connectivity was found in areas and network exerting inhibitory control on nociceptive input like rostral anterior cingulate regions (Gracely et al., 2002; Diers et al., 2008; de Tommaso, 2008; Jensen et al., 2013; Plazier et al., 2015; Truini et al., 2015; Lopez-Sola et al., 2017; Sawaddiruk et al., 2017). In line with these observations, FMS patients showed also abnormalities of functional connectivity in Default mode network (DMN) mainly affecting the referential DMN and the executive control network (Pujol et al., 2014). These changes are similar to those reported in other chronic pain states and are considered to depend on effects of persisting pain on brain (Baliki et al., 2008). Changes in prefrontal. cingulate and insular areas excitability and connectivity were found also to play a role in other relevant manifestation of FMS like affective disturbance and fatigue.

Functional differences in cortical excitability emerged also in a study using TMS of motor cortex in FMS patients. The report showed increased motor threshold, thus a lower corticospinal excitability and reduced short lasting intracortical inhibition (SICI) as well as reduced intracortical facilitation (ICF), that could be related to reduced intracortical GABA and glutamate modulating circuits (Mhalla et al., 2010). Moreover, in a seminal study, Antal et al. (2010) showed that anodal tDCS, can induce a parallel reduction of both SICI and pain perception at VAS scale in FMS patients. Interestingly, other pain states showed a similar condition of reduced SICI and facilitatory stimulation of M1 was able to exert an analgesic effect, restoring also intracortical inhibition (Lefaucheur, 2016).

In agreement with functional changes, also fine structural abnormalities of the gray matter of the same dysfunctional regions emerged from studies using the Voxel Based Morphometry (VBM) technique, that can explore with great detail neuronal density of cortical areas. VBM indeed showed changes in gray matter of prefrontal, insular and cingulate cortex in FMS patients that correlated with intensity of pain, cognitive and affective impairment (Jorge and Amaro, 2012; Jensen et al., 2013; Cagnie et al., 2014; Lin et al., 2016). Taken together these finding seem to delineate a critical network of areas within the pain network that can account for the clinical spectrum of FMS. Indeed, according to a relevant hypothesis, pain and other FMS symptoms can co-occur sharing underlying neural networks (Luerding et al., 2008). Moreover, based on known connectivity of neural structures involved in pain processing and control and on evidence on other pain states, two areas: M1 and DLPFC emerge as optimal candidates for therapeutic neuromodulation. M1 stimulation was found indeed effective for treatment of chronic pain, likely through an inhibitory effect on sensory component of pain while DLPFC, through its connection with limbic system and the DNIC system can exert a modulatory effect on both pain and cognitive and affective symptoms of FMS (Lefaucheur, 2016).

## Methods

### Data Sources and Selection Criteria

We searched three data bases: PubMed, Cochrane library and Scopus until August 1, 2018 for articles published in English with the search terms: “fibromyalgia” and “transcranial” in the field “Title or Abstract.” As concerns tDCS we included only randomized controlled trials, where the diagnosis of Fibromyalgia was made according to the criteria of American College of Rheumatology (1990 or 2010 ACR: Wolfe et al., 1990, 2010). We excluded non-randomized (controlled or open label) papers, single case reports and reviews, papers with patients not meeting ACR criteria for FMS.

Differently, studies based on new tES approaches, other than tDCS, were all mentioned and described even if only those with randomized controlled design were included in the analysis.

Authors BF and BG performed independently the search and selection of the papers and possible disagreement was solved through consultation with a third author (CG).

### Analysis of Risk Bias

Risk bias in the selected studies was explored trough the specifically suited Cochrane tool (Higgins et al., 2011) designed to examine different potential sources of bias: selection bias (random generation sequence and allocation concealment), blinding (subject and assessor), incomplete outcome data (attrition bias), selective reporting (reporting bias), carry over effects (for cross-over trials) and other (not included in the previous categories) bias. Moreover, according to the new author guidelines from Cochrane Pain, Palliative and Supportive Care and the recommendations by Moore et al. (2010), followed by the most recent Cochrane reviews, we included also analysis about two more potential bias sources: “sample size” and “follow-up duration.” The degree of bias risk for such criteria is evaluated according to the thresholds proposed by Moore et al. (2010). As concerns “sample size,” studies with less of 50 participants × arm were considered at high-risk, those between 50–199 at unclear-risk while a low-risk of bias can be presumed for sizes of 200 or more. For the criterium of duration, follow-up less than 2 weeks are considered a high risk, an unclear risk is attributed for periods ranging from 2–7 weeks, while at low risk for bias are considered studies with 8 or more weeks evaluation after the end of stimulation.

**FIGURE 3:**
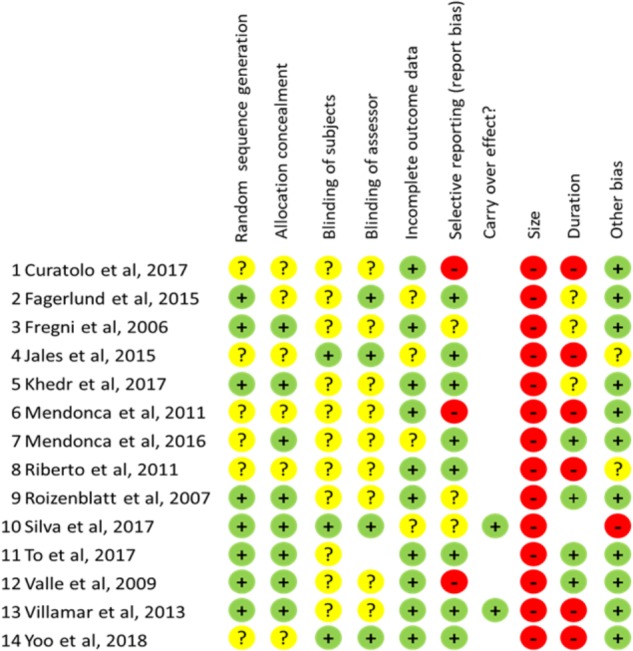
Scores for risk bias analysis for all items evaluated in each study examined. “+”: low risk; “?”: unclear risk; “-”: high risk.

On such a basis, different degree of risk bias (high, unclear, or low) has been attributed for all bias sources to each of the included studies (see Figure 3). Scoring was performed independently by authors DTM and BF and disagreement was solved through consultation with another author (CM).

## Application of tES for Treatment of Fibromyalgia

Fourteen tES studies meeting the selection criteria have been included. The majority of them (12) employed the tDCS technique, (Fregni et al., 2006; Roizenblatt et al., 2007; Valle et al., 2009; Mendonca et al., 2011, 2016; Riberto et al., 2011; Fagerlund et al., 2015; Jales et al., 2015; Khedr et al., 2017; Silva et al., 2017; To et al., 2017; Yoo et al., 2018), while other variants like HD-tDCS, and tRNS have been explored more recently only in 2 randomized controlled studies (Villamar et al., 2013; Curatolo et al., 2017). Given the poor evidence available and the potential of these new techniques, we mentioned, within the tES variants, two more studies that we considered interesting for potential developments even if they don’t meet the criteria for inclusion, one because open label (Castillo-Saavedra et al., 2016) or the other one because exploring only feasibility issues (Carvalho et al., 2018).

### Stimulation Parameters

Two brain area were principally targeted in these studies: M1 and DLPFC that were stimulated alone or compared each-other in the same trial., M1 alone was stimulated in the majority of reports (Mendonca et al., 2011, 2016; Riberto et al., 2011; Villamar et al., 2013; Fagerlund et al., 2015; Jales et al., 2015; Curatolo et al., 2017; Khedr et al., 2017). DLPFC was targeted in the studies by Silva et al. (2017); To et al. (2017), and Yoo et al. (2018). DLPFC and M1 were compared in the studies by Fregni et al. (2006), Roizenblatt et al. (2007), and Valle et al. (2009). A different brain area was targeted in the study by Mendonca et al. (2016), that stimulated supraorbital regions of both sides compared with M1, using an extracephalic reference and exploring only outcome on pain and life quality.

Stimulation intensities of 1.5 mA was employed in the studies by To et al. (2017) and Yoo et al. (2018). Silva et al. (2017), Curatolo et al. (2017), and Khedr et al. (2017) used 1 mA, while 2 mA stimulation was performed in the others (most studies). Stimulation lasted for 20 min in all but one study (Curatolo et al., 2017) where 15 min. tRNS was delivered. Treatment schedule changed significantly across studies. Five daily stimulation sessions were employed by Fregni et al. (2006), Roizenblatt et al. (2007), Fagerlund et al. (2015), and Mendonca et al. (2016). The stimulation period was increased to 10 daily sessions (week-end free) in the reports by Valle et al. (2009); Curatolo et al. (2017) and Khedr et al. (2017). Longer stimulation schedules were used by Riberto et al. (2011) and Jales et al. (2015) (1 week session for 10 weeks) and by To et al. (2017) and Yoo et al. (2018) that delivered 2 session per week for 4 weeks. Single session stimulation was instead performed in the studies by Mendonca et al. (2011), Villamar et al. (2013), and Silva et al. (2017).

All but three studies used cephalic montages with anode on left side (M1 or DLPFC) and cathode on contralateral supraorbital area. Bilateral DLPFC stimulation was performed in two studies with anode on left- in the report by To et al. (2017) or on the right-side in that by Yoo et al. (2018). An extracephalic montage was instead used in the study by Mendonca et al. (2011) comparing M1 vs. supraorbital areas with both anodal and cathodal polarity (see below).

### Outcomes

Pain and impact on life quality, were the most explored outcomes, while less studies examined also effects on other clinical aspects like affective and cognitive symptoms, sleep disturbances and fatigue. In two studies tDCS was explored in add-on with physical exercise with interesting synergic effects (Riberto et al., 2011; Mendonca et al., 2016), while in one (Yoo et al., 2018) authors investigated add-on effects of DLPFC and occipital nerve stimulation (ONS).

Here we separately describe: (1) tDCS studies, further distinguishing by outcomes: effects on pain and on other symptoms (cognitive and affective disturbances, sleep changes, fatigue) and by stimulation sites; (2) tDCS studies in add-on with physical exercise and ONS; and (3) studies on other tES variants like HD-tDCS, tRNS and home based tDCS treatment.

All studies mentioned employing tES for FMS treatment are summarized in Tables 1, 2.

**Table 1:** Transcranial direct currents stimulation (tDCS) studies on fibromyalgia.

Author	Study design	N. Paz	Target	Number of sessions	Aim	Results
Fagerlund et al., 2015	Randomized, sham-controlled.	48 patients	Left M1	Five sessions of anodal tDCS 2 mA, 20 min for five consecutive days.	Relief of pain, stress, psychiatric symptoms.	Anodal tDCS induced statistically significant pain relief. No significant change for the other measures.
Fregni et al., 2006	Randomized, sham-controlled, proof of principle.	32 patients	Left M1 or Left DLPFC	Five sessions of anodal tDCS 2 mA, 20 min for five consecutive days.	Pain relief.	Anodal tDCS of the primary motor cortex induced significantly greater pain relief as compared to sham; the effect was still significant after 3 weeks.
Jales et al., 2015	Randomized, sham controlled trial with CT scan with single photon emission (Brain-SPECT) evaluation.	20 patients	Left M1	10 sessions of anodal tDCS 1 mA, 20 min (once a week for 10 weeks) and brain imaging by Brain Perfusion Scintigraphy.	Pain relief, amelioration of life quality and changes in SPECT imaging.	M1 tDCS was effective for therapeutic pain control and improved quality of life. Significant changes in imaging with decreased biparietal hypoperfusion after stimulation.
Khedr et al., 2017	Randomized sham controlled with evaluation of serum beta-endorphin levels (BEL).	40 patients	Left M1	10 sessions (5 days/week for 2 weeks) of anodal tDCS (1 mA, 20 min). Dosage of serum BEL. Follow-up at 15 and 30 days.	Pain, life quality and mood amelioration and relation with BEL changes by WPI, SS, VAS, pain threshold (primary outcome); HAM-D and HAM-A, serum BEL (secondary outcomes).	M1 tDCS was effective on all outcome measures for pain and mood; BEL increased after treatment (both anodal and sham groups) showing a negative correlation with all other outcomes in the anodal tDCS group.
Mendonca et al., 2011	Randomized sham controlled.	30 patients	Left M1 Left Supraorbital (SO)	One session; 5 groups: M1-anodal; M1-cathodal; SO- anodal; SO-cathodal; sham (extracephalic reference electrode).	To determine current distribution and short-term analgesic effects of tDCS using different electrode montages. Outcomes: VNS, PPT, BD.	SO (both cathodal and anodal) montages, showing at computer simulation current flows through prefrontal cortex, were effective on pain. M1 montages inducing instead temporal current flows was ineffective.
Mendonca et al., 2016	Randomized sham-controlled tDCS treatment in add-on to aerobic exercise.	45 patients	Left M1	Five sessions of anodal tDCS (2 mA, 20 min) for five consecutive days combined with aerobic exercise, 30 min per session.	Pain relief.; anxiety and mood amelioration.	The combination intervention had a significant effect on pain, anxiety and mood.
Riberto et al., 2011	Randomized sham controlled coupled with a physical rehabilitation program.	23 patients	Left M1	Anodal tDCS 2 mA, 20 min. once a week for 10 weeks, combined with multidisciplinary rehabilitation.	Pain relief and life quality.	tDCS add-on treatment showed significantly greater effects on life quality with respect to sham+rehabilitation.
Roizenblatt et al., 2007	Randomized, sham-controlled Bilateral.	32 patients	Left M1 or left DLPFC	Five sessions of anodal tDCS 2 mA, 20 min for five consecutive days.	Pain relief and sleep amelioration.	Anodal tDCS was effective on sleep and pain. M1 treatment increased sleep efficiency and decreased arousals. DLPFC decreased sleep efficiency and increase rapid eye movement (REM).
Silva et al., 2017	Randomized sham-controlled.	40 patients	Left DLPFC	A single session of tDCS 1 mA, 20 min.	Improve alertness, orienting, executive control and pain relief.	Anodal tDCS increased heat pain threshold and tolerance and ameliorated orienting and executive attention. There was no effect on alertness.
To et al., 2017	Randomized sham controlled trial.	42 patients	Bilateral DLPFC Bilateral Occipital Nerve (ON) area	Three tDCS groups: DLPFC, ON, sham. Eight sessions (2 weeks × 4 weeks) of anodal stim. (2 min, 1,5 mA).	Pain and fatigue amelioration.	DLPFC improved pain and fatigue, while ON was effective only on pain.
Valle et al., 2009	Randomized, sham-controlled clinical trial.	41 patients	Left M1 Left DLPFC	Ten daily sessions (Monday–Friday, 2 weeks) of anodal tDCS (20 min, 2 mA).	Pain relief; long lasting effects by longer (2 weeks) treatment.	First evidence that 10 daily sessions give more lasting outcomes. This long-term effect was observed only for M1 stimulation.
Yoo et al., 2018	Randomized sham controlled clinical trial.	58 patients	Bilateral ON Bilateral DLPFC	Three tDCS groups: ON, DLPFC+ON, sham; 8 sessions (2/week × 4 weeks) of anodal stim. (20 min, 1,5 mA).	To explore the add-on effect of DLPFC preceding ON with respect to ON alone stimulation on: disability (FIQ) pain (NRS) and mood (BDI).	ON stimulation was effective on all outcomes measures; DLPFC prestimulation added no further significant effect.


*BD, body diagram; BDI, Beck depression inventory; BEL, beta-endorphins levels; CT, computerized tomography; DLPFC, dorsolateral prefrontal cortex; M1, motor cortex; HAM-D and HAM-A, Hamilton depression and Hamilton anxiety scales; HD-tDCS, high-density tDCS; HS, healthy subjects; NRS, numeric rating scale; ON, occipital nerve; PPT, pressure pain threshold; SO, supraorbital; SPECT, single photon emission computerized tomography; SS, symptoms severity scale; VAS, visual analogic scale; tDCS, transcranial direct current stimulation; tRNS, transcranial random noise stimulation; VNS, visual numeric scale; WPI, widespread pain index*.

**Table 2:** Other tES studies on fibromyalgia.

Author	Study design	N. Paz	Target	Number of sessions	Aim	Results
Carvalho et al., 2018	Randomized, sham-controlled trial., with a specific device for home-based tDCS treatment.	20 healthy subjects (HS). 8 patients	Left M1 in HS; Left DLFC in FMS patients.	Anodal tDCS (20 min, 2 mA) sessions, 10 continuous daily sessions in HS; 5 days (Monday–Friday) for 12 weeks (60 sessions) in FMS patients. tDCS machines specifically suited for home-based stimulation.	Feasibility of home-based tDCS treatment for FMS.	In both groups optimal adherence to the protocol (>90%), good impedance control and general tolerability and safety of the device.
Castillo-Saavedra et al., 2016	Phase II open-label HD-tDCS study.	20 patients	Left M1	At least 15 daily sessions of HD-tDCS.	To establish the number of HD-tDCS sessions required to achieve a 50% pain reduction.	HD-tDCS application maintained for 6 weeks showing a significant and relevant cumulative therapeutic effect. The trial estimate 15 as the median number of HD-tDCS sessions to reach clinically meaningful outcomes.
Curatolo et al., 2017	Randomized, sham-controlled tRNS study.	20 patients	Left M1	10 daily sessions (Monday–Friday, 2 weeks) of tRNS (15 min. 1 mA, 100–600 Hz).	To evaluate effects on pain, cognitive and mood disturbance.	This study is the first evidence about the effect of left motor cortex tRNS on pain, cognitive and mood disturbances in fibromyalgia.
Villamar et al., 2013	Randomized, sham-controlled, crossover HD-tDCS study.	18 patients	Left M1	Single session of anodal, cathodal, and sham HD-tDCS 2.0 mA, 20 min.	Pain relief.	M1 cathodal HD-tDCS stimulation led to significant reduction in overall perceived pain. 30 min after stimulation pain relief was still present cathodal and emerged also for anodal polarity (tardive effect).


*BD, body diagram; BDI, Beck depression inventory; BEL, beta-endorphins levels; CT, computerized tomography; DLPFC, dorsolateral prefrontal cortex; M1, motor cortex; HAM-D and HAM-A, Hamilton depression and Hamilton anxiety scales; HD-tDCS, high-density tDCS; HS, healthy subjects; NRS, numeric rating scale; ON, occipital nerve; PPT, pressure pain threshold; SO, supraorbital; SPECT, single photon emission computerized tomography; SS, symptoms severity scale; VAS, visual analogic scale; tDCS, transcranial direct current stimulation; tRNS, transcranial random noise stimulation; VNS, visual numeric scale; WPI, widespread pain index.*

## tDCS Studies: Effects on Pain and Life Quality

### M1 Stimulation

Fregni et al. (2006), first showed that five sessions of anodal tDCS over left M1 were able to ameliorate pain and life quality in patients affected by FMS, as compared to left DLPFC or sham stimulation (three study groups). The effect was relevant and significant with respect to placebo (58% vs. 30%) and persisted lasting for 3 weeks after the stimulation period. DLPFC stimulation was not effective on pain. but showed a greater (even if not significant) and more persistent effect on depression at Beck depression inventory (BDI) scores. Shortly after, positive effects of M1 stimulation on pain were confirmed by Roizenblatt et al. (2007) by means of the same experimental design. Further studies targeting M1 generally confirmed positive effects on pain and life quality. Using the same experimental design with 5 days session schedule, Fagerlund et al. (2015) reported efficacy of anodal M1 tDCS in FMS patients on measure of pain scores.

Positive effects on pain and life quality were obtained also in other studies that explored longer stimulation periods to induce more lasting effects. Valle et al. (2009) increased the stimulation period from 1 to 2 weeks exploring stimulation of both M1 and DLPFC areas. They confirmed the efficacy of the treatment (significant amelioration of pain symptoms and life quality scores) obtaining also a long-lasting therapeutic effect that remained up to 2 months after the end of the stimulation for M1. Khedr et al. (2017), using the same schedule of 10 days stimulation over left M1, reported significant and persistent therapeutic effects on pain measures (still present at the 1 month follow-up) that correlated with an increase in the levels of serum beta-endorphines. Riberto et al. (2011) tried a study design with different temporal distribution (1 session per week for 10 weeks) to extend the stimulation period and keep the effects of stimulation longer. They found a significant and relevant effect of anodal tDCS on life quality but not on pain, that lasted quite unchanged across all the stimulation period of 10 weeks and was still present at the 1-week follow-up after stimulation. Similar and more extensive results were obtained by Jales et al. (2015), who, using the same temporal design, obtained amelioration not only of life quality score but also of pain measures in a group of FMS patients treated with anodal stimulation as compared to sham stimulation over M1 area. Moreover, using Single photon Emission Tomography (Brain SPECT) authors showed that tDCS was also able to ameliorate (reduce) the biparietal hypoperfusion observed in baseline.

### DLPFC Stimulation

Targeting of DLPFC was generally less effective for treatment of pain in FMS patients. In the three studies comparing M1 and DLPFC, no effects by DLPFC stimulation was reported by Fregni et al. (2006) and by Roizenblatt et al. (2007) while a significant but short lasting (not persistent at follow-up) effect on pain and life quality was showed by Valle et al. (2009). Differently, significant changes in pain scores were reported in two more recent studies. In the single session study by Silva et al. (2017), stimulation of left DLPFC significantly increased heat pain threshold and tolerance. In the trial by To et al. (2017) 8 tDCS sessions over bilateral DLPFC significantly ameliorated pain scores.

### Other Stimulation Sites

The great majority of studies performed anodal stimulation of M1 and/or DLPFC areas all with a cephalic reference. Mendonca et al. (2011) compared stimulation of M1 and supraorbital (SO) region of left and right side with both anodal and cathodal polarities and sham (five stimulation conditions) using an extracephalic reference. They also performed computer simulation to study currents distribution of the different montages within a head model based on tridimensional reconstruction of an MRI scan. Interestingly, they found that SO but not M1 stimulation were able to ameliorate pain in FMS patients. This, however, was not surprising because the study of currents distribution within these specific montages showed flows through the prefrontal areas (involved in the pain matrix) in SO but not in M1 where currents distribution instead principally involved the temporal cortex.

## tDCS Studies: Effects on Other Symptoms (Cognitive and Affective Disturbances, Sleep Changes, Fatigue)

### M1 Stimulation

Less studies explored ability of anodal tDCS to ameliorate cognitive and affective symptoms in FMS patients. In the first report by Fregni et al. (2006) (comparing effects of DLPFC and M1 anodal tDCS) authors found that M1 stimulation, able to ameliorate pain, was instead ineffective on depression. Differently positive effects on anxiety and mood were reported by Mendonca et al. (2016) where tDCS over left M1 was coupled with physical exercise (see below) and in that by Khedr et al. (2017) where M1 stimulation induced significant long-lasting changes at Hamilton anxiety and depression scales (HAM-A and HAM-D) scores that was still persistent at 1 month follow-up.

Differently no effects on cognitive/affective symptoms were reported in the stud by Fagerlund et al. (2015) targeting M1 and in that by Riberto et al. (2011) that explored the effects of M1 tDCS in add-on with a rehabilitative protocol (see below).

### DLPFC Stimulation

In the study by Fregni et al. (2006), comparing DLPFC and M1, DLPFC stimulation, that achieved no significant effects on pain, induced a slight even if not significant changes in the BDI scores. The following year the same group (Roizenblatt et al., 2007), through the same study design, more specifically investigated the effect on sleep disturbances, that are frequent and highly disabling in patients with FMS (Doherty and Smith, 1993), and its correlation with pain improvement. They explored clinical and electroencephalographic (EEG) outcomes and found that anodal M1 stimulation was able to ameliorate sleep in FMS together with a parallel improvement in pain experience. M1 tDCS increased the total sleep time and efficiency, reducing the latency for sleep and REM phase beginning, increasing also the percentage of slow-wave (delta) epochs at EEG that are associated with more effective and restorative sleeping. Differently, opposite effects were achieved by DLPFC stimulation that reduced sleep time and efficiency, increasing latencies for the beginning of sleep and REM phase together with decreasing of delta activity. Such results agree with previous evidences on effects of anodal tDCS over DLPFC in patients with mood disorders, where the positive effect on depression was associated to the increase of arousals and alfa activity at EEG.

More specific cognitive targets were assessed in a very recent study (Silva et al., 2017) that explored the cognitive effects of DLPFC stimulation in FMS focusing more in detail on selective aspects of attention (alerting, orienting, and executive). Interestingly they found that a single session of anodal stimulation had no effect on alertness but was able to improve both pain and orienting and executive attention in FMS patients. Moreover, the effect on attention was found to be independent from that observed on pain.

Effects on fatigue were explored only in one study employing tDCS of bilateral DLPFC (with anode on left side) compared to peripheral occipital nerve stimulation (ONS). Authors showed that targeting of bilateral DLFPC was able to significantly ameliorate fatigue together with pain as compared to ONS that was instead only able to reduce pain (To et al., 2017).

No changes at all by DLPFC stimulation on measures of depression and anxiety were instead found in the study by Valle et al. (2009) comparing DLPFC and M1 stimulation.

## Effects of tES as Synergic Treatment in Fibromyalgia

### Add-On With Physical Exercise

Another therapeutic strategy explored the potential synergic effects of tDCS in add-on to other therapeutic tools. In the study by Riberto et al. (2011) the adding of anodal M1 stimulation to a physical rehabilitative protocol favored a greater effect on pain control. In a recent paper Mendonca et al. (2016) showed that anodal M1 stimulation was able to induce a significantly more consistent effect on pain and life quality if delivered while patient was performing an aerobic physical exercise.

It is known that aerobic exercise can affect a large neural circuit inducing neuroendocrine responses (Schwarz and Kindermann, 1992; Goldfarb and Jamurtas, 1997; Kramer and Erickson, 2007) and other long-lasting mechanism favoring the maintenance of the improvement (Colcombe et al., 2004; Mang et al., 2016; Lulic et al., 2017). Exercise, in fact, modulates the activity in specific cortical regions by learning tools, leading to long-term potentiation mechanisms (Erickson and Kramer, 2009). Mendonca et al. (2016) proved that neuromodulation with tDCS in association with an aerobic exercise training determined a relevant effect on pain, anxiety and mood, probably through a sequential activation and modification of the system by tDCS and exercise, respectively. The same combination approach might have influenced other neural circuits, such as those governing the affective-emotional aspects of pain. Indeed, areas involved in processing of emotions and affective states and more in particular the fronto-limbic network showed impairment in FMS and this could account for abnormal response to pain and for affective disturbance that are both parts of the clinical picture of the disease. This appears worth to mention because the same areas and networks can be modulated by exercise and physical activity that, in turn, has been showed able to ameliorate pain states as well anxiety and depression symptoms (Sciolino and Holmes, 2012; Archer et al., 2014; Kregel et al., 2017).

### Other Add-On Treatments

Another add-on strategy was explored by Yoo et al. (2018) that investigated association of DLPFC stimulation together with ONS and found no further significant advantage of add-on with respect to ONS alone that was effective on measure of pain, life quality and mood.

## Effects of New tES Variants in Fibromyalgia

Besides classical direct current approach, the most till now employed, new application of tES have been recently tried for treatment of FMS. Two recent studies explored the effects of HD-tDCS. Villamar et al. (2013) performed single sessions of HD-tDCS centered over motor cortex (anodal., cathodal, and sham) in FMS patients, showing that both anodal and cathodal polarities were able to induce significant amelioration of pain. The effect started first with cathodal currents and lasted till 30 min after the end of stimulation for both polarities. In another recent open label study with a dose-finding approach, Castillo-Saavedra et al. (2016), explored the effect of long-term HD-tDCS application maintained for 6 weeks, showing a significant and relevant cumulative therapeutic effect (>50% pain reduction and significant amelioration in life quality scores) in one half of the treated patients that persisted across all the stimulation period.

Despite the great interest raised about alternate currents and their potential to interact with the ongoing cortical rhythms and function, no study has until now investigated the therapeutic ability of tACS in fibromyalgia. Our group recently explored the approach of tRNS over motor cortex to treat pain and associated symptoms (cognitive and mood dysfunctions) in patients with FMS. We chose this new approach because the motor cortex was a successful target for the majority of anodal tDCS trials in FMS patients and also because tRNS over motor cortex induced greater facilitation of evoked motor potentials with respect to anodal tDCS (Moliadze et al., 2014; Inukai et al., 2016) showing efficacy not only on pain but also on accompanying mood and cognitive impairment in patients with different pain syndromes (Alm and Dreimanis, 2013; Palm et al., 2016). We treated 2 group of ten patients with real and with sham tRNS, respectively. We evaluated pain, mood and cognitive dysfunctions with a particular focus on the so called fibrofog syndrome that was examined exploring both subjective complains and objective measures of cognitive impairment that characterize fibrofog manifestation, i.e., executive, attentive and working memory performances. After 2 weeks of treatment we observed an extensive therapeutic effect with consistent amelioration of pain and life quality, mood and cognitive measures of fibrofog (Curatolo et al., 2017).

In all these studies, however, patients had to reach Hospitals or Clinical or Research Centers to underwent tES treatment. Differently, in a very recent study on FMS patients, Carvalho et al. (2018) explored the feasibility for home-based tDCS treatment through specifically designed machines equipped with a security control system to guarantee safe application and a software for monitoring stimulation. A group of healthy subjects and one of FMS patients were recruited and trained to use the stimulator. All performed self-stimulation at home through a neoprene cap (easily positioned on the scalp) where preinstalled electrodes were inserted to achieve stimulation of the target area: left DLPFC in FMS patients and left M1 in healthy subjects (HS). Anodal stimulation was delivered (2 mA for 2 min) daily for 5 days a week for a total of 12 weeks (60 sessions) in FMS and continuously for 10 days in HS. In both groups adherence to the study was optimal (more than 90%), electrode impedance (a critical variable to avoid skin lesions) maintained low and very few side effects were reported (not different in type, intensity and severity from those observed in the other tDCS studies).

## Safety and Risk Bias Analysis

### Safety

Transcranial direct currents stimulation was safe and well tolerated in all the studies examined and no serious side effects were reported. The most frequent complaints concerned only itching and tingling sensations that were, however, short-living, vanishing completely in a few minutes after stimulation.

### Randomization and Blinding

All included studies were randomized but randomization criteria were adequately described in 8/14 (57%), leaving a condition of unclear risk bias in the remaining. More concerns are for blinding because strategy for ensure accurate blinding of subjects and assessor are described only in 3/14 (21.5%) and 4/14 (28.6%) respectively, of the studies examined configuring a condition of unclear risk-bias in the remaining. As concerns blinding of subject a critical factor is represented for stimulation intensity, as for currents ≥ 1.5 mA the subject could be able to distinguish between sham and real stimulation. At these intensities indeed, cutaneous sensations could persist across the stimulation period.

### Incomplete Outcome Data and Selective Reporting

The bias risk for incomplete outcome data was generally low as the majority of studies (10/14: 71.4%) correctly addressed this issue reporting opportunely about drop-out level. Similarly, a low bias risk has been found in 8/14 (57%) studies as concerns selective reporting. However, 3 studies (Valle et al., 2009; Mendonca et al., 2011; Curatolo et al., 2017) have been considered at high bias risk because data for size effect calculation at the time points of the study have not been made available by the authors.

### Sample-Size and Follow-Up Duration

All studies examined were based on small samples (less than 20 subjects per arm) configuring therefore a condition of high-risk bias according to the criteria by Moore et al. (2010). Also not appropriate in 6/14 studies (42.8%) was the duration of follow-up because less than 2 weeks or because no evaluation at all was performed to assess the persistence of therapeutic effects.

Generally, the studies performing prolonged stimulation obtained longer lasting therapeutic effect. Ten instead than five stimulation days was able to maintain benefit on pain at one (Khedr et al., 2017) and 2 months follow-up (Valle et al., 2009). Moreover, the strategy to prolong the stimulation period reducing the frequency of the session to one per week was able to maintain the therapeutic effects for 10 weeks in the studies by Riberto et al. (2011) and Jales et al. (2015).

## Discussion

### Summary of the Results

To summarize, the analysis of the tES studies on treatment of FMS evaluated in the review, showed that anodal tDCS of motor cortex, that represent the most studied stimulation target, is able to induce significant therapeutic effects on pain measures and/or life quality in FMS patients, as compared to placebo sham tDCS. Less evidence instead is available about efficacy on other symptoms of FMS (cognitive, affective and sleep disturbances and fatigue) also because these and in particular cognitive dysfunction and fatigue have been generally less explored in the tES studies examined and specially in those targeting M1. Indeed, a few M1 studies reported efficacy on affective symptoms and only one, based on a new tES approach (tRNS) showed therapeutic effects on both motor and cognitive/affective manifestations of FMS (Curatolo et al., 2017).

The other target area studied, DLPFC, was explored only in a few reports and was less effective. A limited, short lasting efficacy on pain and life quality was showed only in other studies (Valle et al., 2009; To et al., 2017) where stimulation time longer than 5 days were used. Differently, positive outcome on attention components was found in the study by Silva et al. (2017). This is partly at variance with studies by rTMS where stimulation of DLPFC showed less effect on pain as compared with M1 site but a greater, more consistent response on mood and cognitive disturbances. The reason for such discrepancies and more in general the pathophysiological mechanisms underlying the effects of tES in the studies examined remain to be delineated. However, concerning the comparison between rTMS and tES about DLPFC effects, a critical general factor could be simply be represented by the very few tES studies exploring the effect of DLPFC and more in particular the outcome on cognitive functions and fatigue. On the other hand, it could not be excluded a greater effect of rTMS on DLPFC at least as concerns affective disorders of FMS, (the ones principally explored) even if this appears less likely due to the proved efficacy of DLPFC tDCS in treatment of resistant and non-resistant depression (Mutz et al., 2018).

Concerning mechanisms underlying the effects of M1 or DLPFC stimulation we have only sparse, direct evidence (trough imaging studies), about activity or connectivity changes induced by the modulation of the target areas and their correlation with the clinical outcome. The study by Jales et al. (2015) found, through brain SPECT imaging, that tDCS treatment on M1 was able to normalize the bilateral parietal hypoperfusion observed at baseline in FMS patient. Cummiford et al. (2016) (report not included in the review because not randomized) studied changes in fMRI resting state and showed, after M1 stimulation, a quite specific pattern of reduced connectivity between thalamus primary motor and sensory areas that correlates with the clinical outcome of pain reduction. This appears in line with the mechanism of anti-dromic inhibitory thalamic modulation that represent one of the ways suggested to explain the analgesic effect of M1 stimulation in different pain states. Differently from M1, no study specifically explored mechanisms underlying the effects of DLPFC stimulation in FMS patients. However, even considering the poor therapeutic evidence available about this target in FMS, one can reasonably infers that the effects of DLPFC stimulation would follow to the known network connectivity of this region. So, the ability of DLPFC to exert antinociceptive effects trough the link with the DNIC system could explain effect on pain, while amelioration of cognitive and affective disturbances can be induced through the connections with the limbic system.

## Limitations and Future Directions

Even if M1 stimulation showed significant ability to ameliorate pain and life quality in FMS in the studies examined, several critical aspects emerge, principally by the risk bias analysis, that reduce the strength of the observed effect. The quality of evidence is indeed hampered by the small patient series investigated, the lack of effective and accurate blinding and the consistent methodological heterogeneity across studies. Moreover, a substantial lack of knowledge remains about the ability to maintain the therapeutic effects over time as only a limited evidence is at moment available about long lasting stimulation protocols. Given to this criticism, anodal M1 tDCS didn’t reach an evidence level to be considered a certainly effective procedure for treatment of FMS. A level B recommendation (probably effective) was indeed provided by a recent consensus paper by the European chapter of the International Society of Clinical Neurophysiology (Lefaucheur et al., 2017) while an even more cautious advice was expressed by a specially suited commission of the European Academy of Neurology. This indeed, based on the method of GRADE (Grading of Recommendations, Assessment, Development, and Evaluation) judged anodal tDCS of motor cortex as still inconclusive for treatment of FMS (Cruccu et al., 2016). A similar critical position was expressed also by the most recent Cochrane review about non-invasive neurostimulation for chronic pain (O’Connell et al., 2018) that, even including more reports with respect of previous evaluation (O’Connell et al., 2014), considered evidence about tES still poor and inconclusive.

Even considering this criticism, tES approach thanks to its safety, ease of use and potential for home-based treatment is worth to be further explored to better define its real therapeutic potential.

Therefore, operational strategies are needed to overcome limitations emerged in the available reports and to exploit the potential of new tES based approaches. To this aim it appears of striking importance to:

(1) Plan adequately powered, randomized controlled trials (20 or more patients × arm) taking care of accurate randomization and blinding and more homogenous methodology, with stimulation periods and follow-up of at least 2 weeks.
(2) Explore, trough appropriately designed RCT studies the ability to maintain long term therapeutic effects through maintenance protocols.
(3) Investigate the potential of new tES stimulation methods like HD-tDCS, tRNS, and tACS, and the add-on combination with other non-stimulation approaches and, last but not least, the generation of new devices for home-based treatment.

All these lines of action are worth to be followed, better through a consensus between researchers, (to ensure opportunely powered and methodologically homogeneous trials) to make the final point about the efficacy of tDCS and other tES variants for treatment of fibromyalgia. This, with final aim to obtain the most effective, extensive, and lasting therapeutic effects through the most easy and inexpensive approach for treatment of FMS patients.

## Author Contributions

FB: work conception, data collection, data analysis, manuscript writing, and manuscript revision. MC, MDT, and PS-P: data analysis, manuscript writing, and manuscript revision. GG: data collection, manuscript writing, and manuscript revision. GB, GC, and BF: data analysis and manuscript revision. All authors contributed to the work and approved paper submission.

## Conflict of Interest Statement

The authors declare that the research was conducted in the absence of any commercial or financial relationships that could be construed as a potential conflict of interest.
